# Perceived Constraints on Active Recreational Sport Participation among Residents in Urban China

**DOI:** 10.3390/ijerph192214884

**Published:** 2022-11-12

**Authors:** Lan Lin, Qun Liu, Xiao Xiao, Qin Luo

**Affiliations:** 1 School of Geographical Sciences, Institute of Tourism, Fujian Normal University, Fuzhou 350117, China; 2 School of Community Resources and Development, Arizona State University, Phoenix, AZ 85004, USA

**Keywords:** perceived constraints, leisure opportunities, active recreational sport participation, urban residents, China

## Abstract

Recreational sport participation is an important pathway to improving the quality of life. While facilities for recreational sports are provided in many urban areas in China, how urban residents might be aware of or use these facilities for recreational sport participation is still a vague notion in the literature. This study explored the linkages between perceived leisure constraints and active participation in recreational sports among urban residents. We collected data samples from 2901 urban residents in China to identify their perceived constraints and the effects of the perceived constraints on active recreational sport participation by structural equation models. Five perceived constraints of active recreational sport participation were identified: intrapersonal, interpersonal, environmental condition, facility-service management, and leisure opportunity constraints. More specifically, leisure opportunity, intrapersonal, facility-service management, and interpersonal constraints were the four most important constraints limiting active recreational sport participation of urban residents. Theoretical and practical implications to facilitate the active recreational sport participation of Chinese urban residents were discussed.

## 1. Introduction

Over the past two decades, the inactive and inadequate participation in recreational sports have been a challenging issue for the urban residents’ quality of life. Urban areas should provide a dwelling, work, transportation, and recreation functions to satisfy the urban residents’ diverse needs [1]. Meanwhile, the fundamental role of people-oriented functions of a city rather than the material-based or political power-based functions is highlighted in the modern urban development agenda. Therefore, the concept of “leisure cities” and “healthy cities” has been widely promoted and implemented in many urban areas in China [2,3,4]. Through these initiatives, the diverse culture and lifestyles of urban residents are valued, which empowers urban residents to freely choose health-friendly leisure behaviors, and guarantees the physical fitness and leisure rights of urban residents through various urban development and revitalization processes.

Inspired by the national strategy “Healthy China” initiated in 2017, a large number of recreational facilities for physical exercise, leisure space, and service management were developed to encourage mass sports and fitness at the national level. Nowadays, many cities in urban areas have built varying levels of physical exercise facilities and leisure resources. Based on the statistics of the Sixth National Stadium Census Data Bulletin (2013), there are 82 types of sports complexes in total in China, with 1,694,600 sports venues; the number of sports venues in urban areas is 96,270 with an area of 1.337 billion square meters. Consequently, participating in physical activities during one’s leisure time has become one of the primary leisure activities for urban residents. Among all types of leisure activities, recreational sport is one of the most popular leisure activities in China.

Recreational sporting activities are leisure activities that combine both sport and recreation. These activities fall under the umbrella of play [5], and do not have to be competitive or require particular equipment or rules [1,6,7]. A number of studies have examined the positive outcomes of the physical activity component of recreational sports and reported that recreational sport activity enhances circulatory health, provides mental clarity, promotes successful aging, and promotes social interaction [8,9,10,11,12]. However, the benefits of recreational sport participation vary between rural and urban areas [13].

While urban areas have provided the resources for recreational sport, urban residents might encounter constraints when participating in recreational sports. Inadequate facilities, lack of leisure time, and lack of knowledge are common constraints for urban residents to engage in urban recreational sport [14]. In China, the leisure activities of urban residents are highly constrained by socio-economic resources and time [15]. Crawford and Godbey’s [16] framework of leisure constraints has discussed the link between perceived constraints and non or less leisure participation, and the framework has been widely applied to different types of recreation settings. Moreover, leisure activity participation might be different among social groups, as racial/ethnic minority groups, women, adolescents [17,18], and people with disabilities were traditionally disadvantaged groups in leisure participation. However, most leisure constraint studies have focused on specific group/populations in North America [19,20], Europe, and Australia, and the content are constraint determinants for the level of participation in leisure time physical activity (LTPA) in general [21,22,23,24]. The application and extension of this framework research to recreational sport participation are limited [25,26]. From the theoretical perspective, very few studies have examined the perceived constraints of urban residents for active recreational sporting participation in China. The urban population is growing rapidly in China, accounting for nearly two-thirds of the population, and more and more residents actively participate in recreational sports activities. Active recreational sport participants have been the main users of urban leisure sports venues and facilities. Whether the recreational facilities and leisure resources could meet the urban residents’ recreational sport activity needs is an emerging question for park managers and urban designers. To better explore recreational sport, academics and professionals must understand and appreciate the perceived constraints facing urban active recreational sport participants. Specifically, the study aimed to (a) identify the perceived constraints affecting active recreational sport participation; (b) test the relationships between perceived constraints and active recreational sport participation, and (c) examine the differences in the perceived constraints and active recreational sport participation by gender. We tried to test and extend the leisure constraints model [16] in non-Western contexts, which not only seek to understand the choice determinants of the active participant, but also to comprehend the range of perceived constraints preventing active sport participants from becoming inactive or non-sport participants in urban China.

## 2. Literature Review

### 2.1. Recreational Sport Participation in Urban Area

Recreational sport activities are typically conducted during leisure time and are an unstructured means of entertainment [27,28,29], which is valued as a way to improve an individual’s quality of life [25]. Recreational sport participation can bring benefits to individuals from both physiological and psychological perspectives. Physiologically, regular participation in recreational sports might promote health including reducing the risks of cardiovascular disease [30], diabetes [31], and extending the length of life [30]. Psychologically, the social interactions of recreational sport participation can enhance the individuals’ self-esteem and facilitate positive emotions [32]. These benefits were manifested in studies among different regions and across time, for example, a longitudinal study found that regular recreation sport participation could improve fitness, develop healthy lifestyles, enhance mood, and strengthen attitudes toward fitness [33]. A few pilot studies have examined the scales of recreational sport participation and found that the participation was a multi-facet process that involves: (1) challenge, (2) victory, (3) sensory, (4) workout, and (5) social [34]. In addition, recreational sport participation levels might be different among socio-demographic groups. Gender has been one of the most important socio-demographic factors that lead to disparities in recreational sport participation levels, and females are more likely to encounter constraints in participating in recreational sports than males in general [25,35]. Perceived constraints are not only barriers that might influence motivation, but also as potential motivators for participation [36,37].

Recreational sport participation also varies greatly between urban and rural areas. For instance, a recent study found that rural residents were less interested in recreational sports than urban residents. Similarly, a study examined factors associated with youth sport participation and found that children in rural communities experienced more constraints of sport participation than those living in urban communities [38]. Chick et al. [39] found that older people listed constraints related to age and health more frequently than younger people while younger informants were more concerned with resources such as money in the urban Taiwanese.

Recreational sports, as an important pathway to facilitate mass sports and healthy cities in China, have been highlighted in the recent National Agenda. Providing sports facilities, organizing public sports activities, and enhancing the awareness of physical exercises can facilitate engagement of recreational sport participation for the general public [40]. The participation of Chinese urban residents in sports activities is spatially clustered in recreation areas and facilities such as parks, neighborhood gardens, and urban squares [41]. Although recreational sports participation is encouraged at the local, regional, and national levels, research on the participation of Chinese urban residents in recreation sport is still in a notably nascent stage, and more research is needed to examine the perceived constraints for the active participation of urban residents in recreational sports in China.

### 2.2. Leisure Constraints among Urban Residents

Leisure research in Western countries is one of the oldest domains of social science research, which has a long history of more than 130 years. Since Crawford and Godbey put forward the concept of leisure constraint in 1987, which has been widely assumed in the constraints literature, that the constraints people perceive lead either to nonparticipation or to reduced leisure participation. Constraints have been defined as “the factors that are assumed by researchers and perceived by individuals to inhibit or prohibit participation and enjoyment in leisure” [42] with additional possibilities to enable leisure [36]. Scholars generally agree that the process of people striving for leisure rights is also a process of overcoming various leisure constraints.

Constraints occur at the intrapersonal, interpersonal, and structural levels that operate in a hierarchy [16,36], which was subsequently expanded by Crawford et al. [43]. Since that time, many researchers have debated the scholarship on leisure constraints and noted possible interactions between intrapersonal, interpersonal, and structural constraints [44,45,46]. Intrapersonal constraints are the individuals’ characters, values, and personalities that prohibit the participation in recreational sports [16] such as a lack of skills and interests, stress, and health concerns [47,48]. Interpersonal constraints, however, stem from interactions and relationships with social groups such as a lack of companions, family support, or encountering discrimination from other leisure participants [32]. Structural constraints are external factors intervening in recreational sport participation such as socio-economic resources, weather, and work schedule. Findings on the impacts of the three types of constraints on recreational sport participation are mixed in earlier studies [32], while a few studies have suggested that intrapersonal constraints seemed to be the most powerful predictors of commitment to recreational sport participation [25,37,49]. 

Although considerable research efforts have been focused on examining leisure constraints in the Western context, research on the leisure constraints in the non-Western context is at a notably nascent stage, in particular, recreational sport constraints in urban China. Dong and Chick [15] examined leisure constraints for urban residents in six cities in China and found that a lack of time and money were the primary constraints for leisure participation of Chinese urban residents. Chick et al. [39,50,51] found that cultural consonance can be a moderator between leisure constraints and leisure satisfaction for urban residents in China. Liu and Walker [52] examined the effects of urbanization, motivation, and constraint on the participation of Chinese people in leisure time physical activity and found that structural constraint was the most important constraint, followed by interpersonal barrier. On the other hand, perseverance was the most motivation positively influenced LTPA, followed by enjoyment. Moreover, Chen [53] and Lin et al. [54] noted that spatial, social, and structural constraints (e.g., distance to facilities/venues and lacks of time) were key factors influencing Chinese people’s LTPA.

### 2.3. Extended Three Dimensions of Leisure Constraints

Although the three types of leisure constraints have been applied and tested widely in earlier studies, a few studies have highlighted the concern that constraints may not easily be delineated into a categorization of intrapersonal, interpersonal, and structural constraints [46,55,56,57,58,59]. Other researchers have also noted possible interactions between intrapersonal, interpersonal, and structural constraints [44,59,60]. Some researchers have investigated how combinations of personal characteristics (e.g., motivation, gender, age, socio-economic status, and race/ethnicity) affected the individuals’ constraints to outdoor recreation [45,61,62,63]. Alexandris et al. [37] provided evidence that motivation acts as an intervening variable between constraints and recreational sport participation. A few recent studies have identified additional factors impacting leisure participation rather than the traditional three-dimension approach. Leisure-related facilities and service management, leisure opportunities, and cultural consonance are important factors associated with leisure participation. For example, Perry et al. [64] found that the lack of transportation facilities was an important constraint for racial/ethnic minority groups to participate in leisure activities in national parks. Leisure opportunities or initiatives such as more efficient and accessible transportation facilities and programs can facilitate leisure participation for marginalized groups [63,64]. However, the coherent impacts of these factors with the three dimensions of constraints on recreational sport participation have not been fully documented in earlier studies, particularly in the context of urban areas in China. There is still limited understanding of cultural variation in leisure constraints [15,50,65,66,67]. Perceived constraints on recreational sport participation requires more cross-cultural and comparative research.

## 3. Materials and Methods

### 3.1. Hypotheses and Theoretical Model

**Figure 1:**
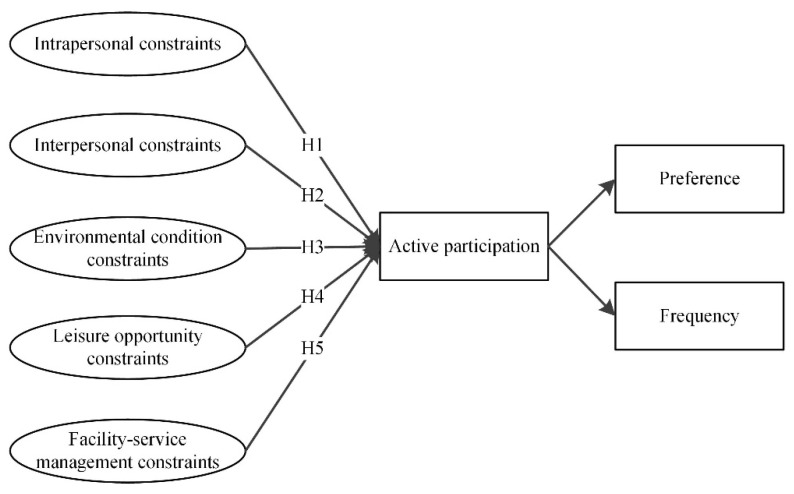
Hypothesized perceived constraints and the active recreational sport participation model.

Drawing on leisure constraints theory [16,43,47] and previous leisure constraint research, a hierarchy model of leisure constraints proposes that intrapersonal, interpersonal, and structural constraints influence the individual’s preferences for leisure activities and participation coherently. Building on the hierarchy model of leisure constraints and additional factors associated with leisure participation identified in earlier studies, we propose a five-category hierarchical model of recreational sport perceived constraints. Two categories were referred to as intrapersonal and interpersonal perceived constraints [27,28]. Structural perceived constraints were divided into three sub-dimensions: environmental, leisure opportunity, and facility-service management [59]. We first proposed an initial theoretical model and posited that all five perceived constraints would influence the individuals’ preferences and frequency of active recreational sport participation. In order to test the multi-variate relationship between the five perceived constraints and active recreational sport participation in leisure time, five hypotheses were stated in the following and are presented in Figure 1.

**H1:**  *The intrapersonal constraints would have a negative relationship with active recreational sport participation*.

**H2:**  *The interpersonal constraints would have a negative relationship with active recreational sport participation*.

**H3:**  *The environmental condition constraints would have a negative relationship with active recreational sport participation*.

**H4:**  *The leisure opportunity constraints would have a negative relationship with active recreational sport participation*.

**H5:**  *The facility-service management constraints would have a negative relationship with active recreational sport participation*.

### 3.2. Perceived Constraints

Constraints were measured by modified variables from Hubbard and Mannell [27], White [28], and Zhu et al. [59] to make the content aligned to the recreational sport activities in the study locations. Participants were asked to report on the degree of agreement with 23 items of perceived constraints for active recreation sport participation. The 23 items included physical problems, psychological problems, negative attitudes toward fitness, lack of interests, not skilled enough, lack of partners, negative attitudes of family and friends, lack of coaches’ guidance, bad weather, fear of safety, poor hygiene, limited opening hours, lack of time, lack of fitness fees, fitness activities are limited, fitness fees are too high, inaccessibility, unattractive programs, lack of personalized services, lack of professional management, inadequate programs and facilities, lack of information, and environment-unfriendly. A five-point scale (1 = “strongly disagree”, 2 = “disagree”, 3 = “neither agree or disagree”, 4 = “agree”, 5 = “strongly agree”) was used to measure the respondents’ agreement with the perceived constraints. A principal components analysis was conducted to identify the dimensions of the perceived constraints.

### 3.3. Recreational Sport Participation

Recreational sport participation was measured by 10 activities based on the Sixth National Stadium Census Data Bulletin (2013). The Sixth National Stadium Census Data Bulletin is a nationwide survey on sport participation for Chinese residents, which was commissioned from 2011 to 2014. The ten activities included team sports (e.g., football, badminton, and basketball); fitness-related activities (e.g., aerobics, dancing); and individual activities (e.g., swimming, yoga, walking, and jogging). Respondents were asked as to which recreational sport activities they always participated in during their leisure-time, then answered the preferences and frequency for the activity that influenced the desire to active participate. In this study, all of the respondents were asked to rate two statements by preference for (e.g., 1 = “not like at all”, 2 = “like it slightly”, 3 = “like it moderately”, 4 = “like it a lot”, 5 = “like it extremely”) and the frequency of participation (e.g., 1 = “once to twice times per month”, 2 = “one time per week”, 3 = “ twice to three times per week”, 4 = “four to five times per week”, 5 = “more than 6 times per week”).

### 3.4. Data Collection

The survey was conducted by members of our research team. Our research object was to investigate the perceived constraints of active participation. The samples recruited in the study consisted of citizens who had actively participated in recreational sport activities. A self-administered, on-site questionnaire survey was distributed randomly in both public physical exercise places and private physical fitness clubs in three Chinese cities (i.e., Nanjing, Fuzhou, and Xiamen). Nanjing, Fuzhou, and Xiamen are important pilot cities for national sports promotion, and residents in these three cities have relatively higher levels of awareness about recreational sport participation, making them rational and representative for a recreational sport constraints study. The survey was administered across both weekends and weekdays from July 2014 to August 2016. 

The questionnaire included three main parts: the behavioral characteristics of active recreational sport participation (i.e., degree of preference and participation frequency), perception of the constraints in recreational sport participation, and demographic information (e.g., gender, age, lifestyle, occupational status, income, and education). Overall, 2927 citizens participated in the study, and 99.11% of them completed the questionnaire (N = 2901). The sample sizes from Nanjing, Fuzhou, and Xiamen were 926, 1049, and 926, respectively.

## 4. Results

### 4.1. Demographic Characteristics of the Sample

Of the 2901 sample, 35.3% were females and 67.7% were males. As shown in Table 1, the age profile was mixed among various age groups including people aged 46 to 65 (14.8%), 36 to 45 (21.4%), 26 to 35 (36.7%), 19 to 25 (20.2%), and less than 18 (6%). For the education background, 45.5% of the respondents had an undergraduate degree and 23.9% had junior college qualifications, respectively. Personal monthly income primarily fell into the classifications of lower than 3000 yuan (20.8%) and from 3001 to 5000 yuan (33.6%). In terms of occupation, 30.1% of the respondents were working at state-owned enterprises, and 27% were working at government-sponsored institutions. Students and public functionaries were 13.4% and 12.1%, respectively.

### 4.2. Principal-Component Analysis

A principal component analysis was conducted to categorize the 23 items related to the recreational sport participation scale. Cronbach’s alpha was used to test the reliability and internal consistency, and the values of KMO and Bartlett’s spherical were used to test for sampling adequacy. Results showed that the Cronbach’s α of the whole scale was 0.95. The value of KMO was 0.96, which was greater than 0.7. Bartlett’s spherical test value was 43,573.25, which reached a significant level under the condition of 253 degrees of freedom and *p* < 0.001. The data were suitable for principal component analysis (PCA).

The PCA was conducted by the SPSS 17.0 Factor program and AMOS 22.0. Only those components with an eigenvalue greater than 1.0 were retained and rotated, and both orthogonal and oblique rotations were used. After 66 times of factor-analysis, it indicated that the six initial constraint items with factor loadings were less than 0.7 and deleted. The PCA was employed again for the remaining 17 items, and five factors emerged to account for 74.87% of the variance (Table 2). The Cronbach’s α of the whole scale was 0.91. The value of KMO was 0.93, which was greater than 0.7. Bartlett’s spherical test value was 30,631.49, which reached a significant level under the condition of 171 degrees of freedom and *p* < 0.001. The internal consistency reliability of the whole scale was 0.83. All items proved to have acceptable internal consistency reliability and validity. The five extracted factors were defined as follows: intrapersonal constraints, interpersonal constraints, environmental condition constraints, leisure opportunity constraints, and facility-service management constraints.

### 4.3. Confirmatory Factor Analysis

The validity of the hypothesized measurement model was tested in the AMOS 22.0 using confirmatory factor analysis (CFA), followed by a test of the hypothesized structural relationships. Results of the CFA demonstrated that the measurement model was an acceptable fit to the data (χ^2^ = 939.83, df = 109, ρ < 0.01; RMSEA = 0.05, 90% lower = 0.05, 90% upper = 0.05; CFI = 0.97; NNFI = 0.97). A full summary of the CFA results including the means, standard deviation, standardized factor loading, bootstrapped standard error, critical ratio, average variance explained, and composite reliability were shown for each of the variables in Table 3. The mean score for all perceived constraints indicated that urban residents rated the intrapersonal constraints as the most important perceived constraint (M = 4.02) that limits the active recreational sport participation. Interpersonal constraints were rated as the second important perceived constraint (M = 3.75), followed by environmental condition constraints (M = 3.75) and facility-service management constraints (M = 3.70). The leisure opportunity constraints had the lowest mean of 3.67 among the five perceived constraints. The variables in the intrapersonal constraints dimension ranged from 3.83 to 4.23, and the lack of interests and physical problems were the most important factors in this dimension. Poor hygiene and negative attitudes toward fitness of the two constraint subscale scores were also higher, which were rated as important reasons for active participation. For the preferences for active participation, respondents were generally likely to participate in recreational sport activities a lot (M = 4.19). The average frequency of active participation was 3.57, which indicated that urban residents wished to participate in recreational sport activities twice to three times to four to five times per week.

Measurement model constructs indicated acceptable reliability with values of the Cronbach’s alpha (e.g., α ≥ 0.7) calculated for each item, and ranged from 0.74 to 0.91. Campbell and Fiske (1959) stressed a successful evaluation of discriminant validity that showed that a test of a concept is not highly correlated with other tests designed to measure theoretically different concepts. Test statistics and composite reliability were also acceptable (ρ ≥ 0.7), and the average variance explained was validity (AVE ≥ 0.5). In Table 4, the discriminant validity was acceptable for all of the latent constructs (less than 0.85).

### 4.4. Structural Equation Models

In this study, we followed a two-step modeling procedure for structural equation modeling (SEM) to test the hypothesized measurement model. The 17 measuring perceived constraint scales obtained from PCA were the observed variables in the SEM. Intrapersonal constraints, interpersonal constraints, environmental condition constraints, leisure opportunity constraints, and facility-service management constraints were the latent variables of cause in the SEM. The preference and frequency of active participation were treated as latent variables of SEM. Figure 1 shows the five hypothesized paths between the perceived constraint latent factors and active participation latent factors, followed by a test of the hypothesized measurement structural relationships for latent variable modeling. 

The SEM model fit was judged by using multiple fit indices, and widely-used indices including the chi-square (χ^2^)/degree of freedom ratio, normed fit index (NFI), comparative fit index (CFI), non-normed fit index (NNFI), and root mean square error of approximation (RMSEA). The chi-squared test is biased against sample size and model complexity. While a non-significant value of chi-square/degree of freedom ratio suggests a good fit to the data, the statistics are known to be highly sensitive to sample size. When the sample size is greater than 200, the chi-square/degree of freedom ratio is often not used as an insightful index to test the goodness-of-fit for the SEM model. The indices of NFI, CFI, and NNFI should be greater than 0.9, and the RMSEA value of less than 0.08 also indicates an acceptable model fit. These criteria were used in assessing the fit of our models. Considering the relatively large sample size of our SEM model (N = 2901), we used NFI, CFI, NNFI, and RMSEA as the goodness-of-fit indices for the SEM model.

We first tested the fit indicators of the initial measurement model. In Table 5, the results of the initial measurement model indicated a poor goodness-of-fit (GFI = 0.76, RMSEA = 0.12, AGFI = 0.69, NFI = 0.78, CFI = 0.79, IFI = 0.79), which meant the initial measurement model needed to be modified by correlating a new structural relationship among the error variables and using the modification indices. All indices showed a high goodness-of-fit of the revised SEM model (GFI = 0.96, RMSEA = 0.05, AGFI = 0.95, NFI = 0.97, CFI = 0.97, IFI = 0.97). A summary of the overall fit indices for the initial measurement model and the final structural model is presented in Table 5.

**Figure 2:**
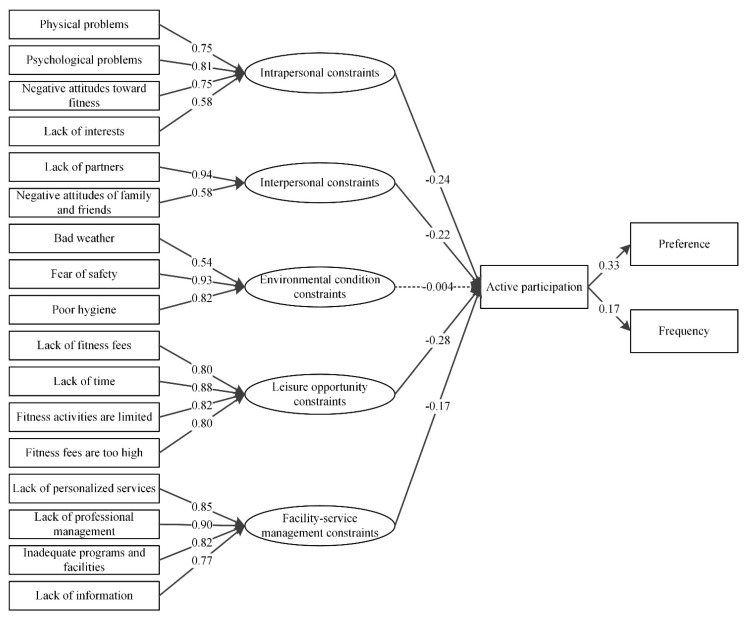
Graphical summary of the structural model results. Dotted line indicates the non-significant results.

The test of the final structural model also revealed that the standardized parameter coefficients were significant and the data supported all of the hypotheses except for Hypotheses 3 (Table 6). The leisure opportunity constraints had a direct negative effect on active participation (S.E = 0.01, *p* < 0.001) and was the strongest factor impacting active participation in the SEM. Intrapersonal constraints also had a direct negative relationship with active recreational sport participation (S.E = 0.02, *p* < 0.001) (Figure 2). The facility-service management constraints had a negative impact on active participation (S.E = 0.02, *p* < 0.001). and the interpersonal constraints had a negative impact on active recreation sport participation, while the impact was weaker compared to the other three factors. However, we did not find support for the hypothesized relationship between the environmental condition constraints and active participation.

### 4.5. Multi-Group Comparisons

To examine the generalizability of the hypothesized SEM to multiple socio-economic factors, we conducted multi-group comparisons of SEMs for active recreation sport participation. We divided the sample to two groups: female respondents (n = 1023) and male respondents (n = 1878), and tested the SEMs between the two groups. We tested the SEMs using six types of models including unconstrained, measurement weights, structural weights, structural covariances, structural residuals, and measurement residuals, and selected the model with the highest levels of goodness-of-fit. Among the six models, the structural residual model was selected as the final model since it had the lowest AIC and ECVI. In the final SEM model, all goodness-of-fit indices met the standards of the SEM (Table 7).

**Figure 3:**
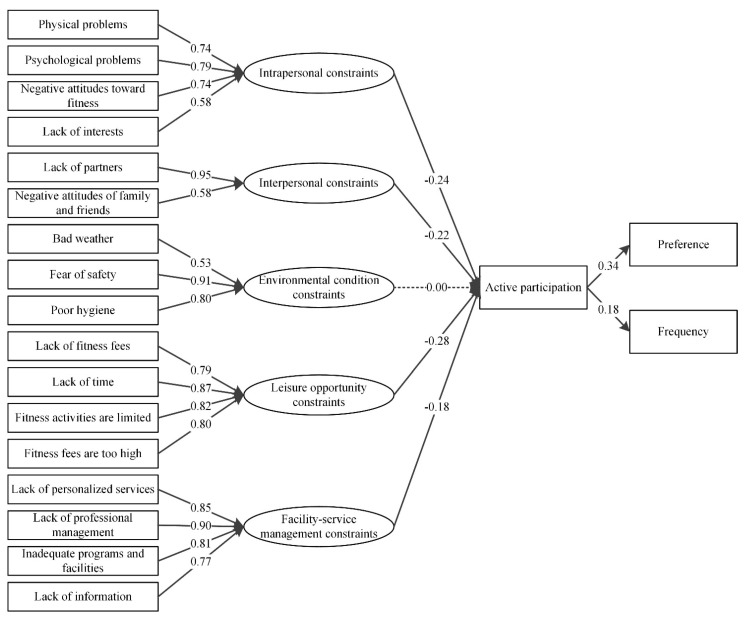
The path of standardized estimates for male groups in the structural residuals model.

**Figure 4:**
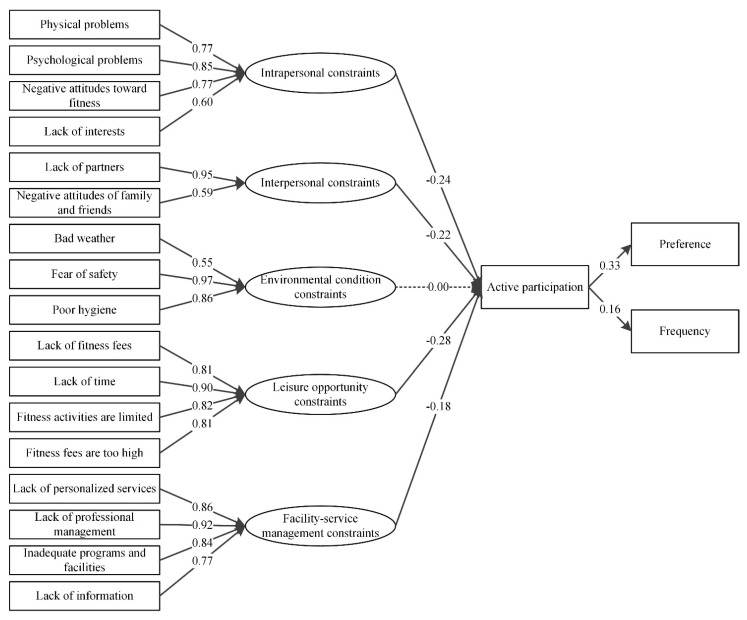
The path of the standardized estimates for female groups in structural residuals model.

The standardized coefficients of SEM for males and females are shown in Figure 3 and Figure 4. In general, the results indicate that the SEM model can be accepted by both female and male groups, and no significant differences were detected for the standardized coefficient between two groups. The leisure opportunity constraints, intrapersonal constraints, facility-service management constraints, and interpersonal constraints were also the four most important perceived constraints for the male and female residents. 

## 5. Discussion and Conclusions

This study analyzed the perceived constraints of active recreational sport participation and tested the relationships between the perceived constraints and active recreational sport participation by urban residents in China. The findings provide support and expansive clarification of a conceptual model of leisure constraints [43], and also provide theoretical and practical implications to encourage recreational sport participation by urban residents in China. Theoretically, our study findings support the model of leisure constraints, and extend the Crawford’s leisure constraint model (1991) to broader dimensions by highlighting the importance of leisure opportunities constraints, intrapersonal constraints, facility-service management constraints, and interpersonal constraints in the urban residents’ active recreational sport participation. Aligned with Crawford’s leisure constraint model [43], the structural constraints play an important role on limiting recreational sport participation, however, the effects of structural constraints on active recreational sport participation were primarily manifested in two sub-order dimensions in our study: leisure opportunity constraints and facility-service management constraints. The SEM model indicates that leisure opportunity constraints such as lack of time, fitness activities, or expensive fitness fees, could be the most significant factors that prohibit the active participation of urban residents in recreational sport activities. This result is aligned with findings in earlier studies that leisure opportunities and initiatives can mitigate the constraints for leisure activities, particularly for traditionally marginalized groups [63,64]. Urban areas, particularly urban areas in China, often have a high density of population and condense residential and industrial construction, making the leisure opportunities essential for urban residents to be able to or willing to actively participate in diverse recreational sport activities. Notably, this result highlights the necessity of leisure opportunities (e.g., perceived freedom, not busy, have free time, and multiple choice of fitness consumption) for active recreational sport participation by broader urban residents. As noted in earlier studies, developing second-order factors with the traditional three-dimensional (intrapersonal, interpersonal, and structural constraints) framework can be helpful in expanding the theory of leisure constraints [48]. Our study extended the generalizability of leisure constraint theory by developing five-dimensions perceived constraints and particularly tested the validity of the sub-dimensions by active recreational sport participation. The multi-group comparisons of SEMs indicate that the five-dimensional perceived constraints also exist in both female and male groups, and the same four constraints impacting active recreational sport participation can be generalized for female and male residents in urban China. 

Aligned with Crawford’s leisure constraint model [43], the SEM model suggested that intrapersonal and interpersonal constraints would limit the urban residents’ active participation in recreational sport activities. For intrapersonal constraints, psychological problems and negative attitudes toward fitness were the most important perceived factors limiting active recreation sport participation, indicating that the psychological or cognitive issues, rather than the physical problems, prohibit the urban residents’ active recreation sport participation to a large extent. For interpersonal constraints, the SEM model indicates that the lack of family support or partners can affect the active participation of urban resident’ in recreational sports, however, the effect was minimal compared to the intrapersonal constraints. This finding is aligned with the findings in Godbey et al. [56], where the impacts of the interpersonal and intrapersonal constraints on leisure participation were mixed among different recreational settings. Social constructivism holds the view that the self is a relationship that is associated with a certain history and culture. Under the long-term influence of traditional Chinese culture characterized by Taoism and Confucianism, Chinese people have a sense of collective and relationship-oriented self. They are mostly influenced by others such as family and friends (i.e., Markus and Kitayama’s (1991) [68] self-construal findings also stated that while Westerners were more likely to endorse an independent self (e.g., to value being unique), East Asians were more likely to endorse an interdependent self (e.g., to value fitting in). Our findings echo some researchers’ findings (e.g., Chick et al. [50,51,52]; Liu and Walker [52]; Liang and Walker [69]) that cultural consonance can be a moderator between leisure constraints and leisure participation. For urban residents in China, interpersonal constraints may play important factors in indigenous Chinese concepts (e.g., collectivism; the effect of family and friends; social status and prestige).

Our study findings also suggest that facility-service management constraints can significantly limit the active participation of urban residents in recreational sport activities. Rather than the traditional leisure activities that rely on natural resources (hiking, mountain biking, fishing), recreational sport activities are highly dependent on the facilities and services, particularly indoor sporting activities. Inadequate and unprofessional management of the facilities, services, and information can be essential perceived barriers for the active participation of urban residents in recreational sport activities. The facility-driven nature of recreational sport activities highlights the need to add the specific dimension of facility-service management to overcome the urban residents’ perceived constraints for active sport participation.

## 6. Management Implications

Our study findings also provide insightful guidance and management strategies for recreational sports managers and stakeholders. First, to address the leisure opportunity constraints, government agencies, and/or sport organizations could implement practical strategies such as providing more flexible operational schedules for sport activities, increasing a variety of personalized, engaging, and affordable sports leisure activities as well as promoting the strategic establishment or expansion of leisure activities and venues to encourage Chinese urban residents to actively participate in sport activities more frequently. From the perspective of intrapersonal constraints, inspiring the urban residents’ enthusiasm for positive participation, enhancing the awareness about healthy lifestyles, and encouraging long-term commitment for physical exercise can be possible ways to increase active recreational sport participation. 

Our results also indicate the importance of facility-service management in active recreational sport participation for Chinese urban residents. The inadequate management for recreational sport facilities and services may be related to the current priorities on material leisure space construction rather than service management quality in the past ten years. Based on our study findings, improving the environmental management levels, service quality, and attractive fitness programs and the convenience of fitness information would help attract more residents to active engage in recreational sport participation in China. This included specific elements such as

- Increasing the professional service capabilities of employees;
- Increasing the professional quality of coaches and their interpersonal communication skills;
- Promoting a personalized, diversified, and convenient service experience;
- Improving a harmony and user-friendly atmosphere that is full of mutual assistance and comfort;
- Building a leisure service information network for venue booking, fitness guidance, physical exercise analysis, physical fitness monitoring, and communication of recreational sport participation.

Moreover, “having a companion who exercises together”, followed by “proactive support attitude of family (friends) to participation” are tackling strategies to overcome perceived constraints for active sport participation. 

## 7. Limitations and Future Research

The study assessed the perceived constraints of active recreational sport participation in a statewide random sample of Chinese urban residents and also provided support for the four hypothesized relationships between the perceived constraints and active recreational sport participation. There were several study limitations. Future research with populations and activities in different cities would be needed to determine the universality of the findings identified. Furthermore, future studies might address the impacts of the COVID-19 pandemic on the constraints of recreational sport participation if the newest dataset is available. 

Second, the construct structure is often believed to be ambiguous due to the intertwining relationships among leisure constraints [57]. Although this structural model is useful for analyzing the mutual and multiple relationships between the perceived constraint variables and active leisure participation, sample statistics could be produced that were poor estimates of the true population parameters. Our sample size (2901) to estimated parameters (36) ratio was 80.6 to 1, which was well above the minimum power needed for model estimation cited by structural equation modeling experts. We need to consider the sample size for the validation in future research.

## Figures and Tables

**Table 1:** The demographic characteristics of the sample.

Variables	Category	Number	Percentage
Gender	Man	1878	64.7
	female	1023	35.3
Age	≤18	173	6.0
	19–25	586	20.2
	26–35	1064	36.7
	36–45	620	21.4
	46–55	323	11.1
	56–65	108	3.7
	>65	27	0.9
Education	Junior high school or lower	176	6.0
	High school/Technical secondary school	405	14.0
	Junior college	693	23.9
	Undergraduate	1320	45.5
	Postgraduate	307	10.6
Personal monthly income (RMB)	≤3000	603	20.8
	3001–5000	976	33.6
	5001–7000	505	17.4
	7001–10,000	440	15.2
	≥10,001	377	13.0
Occupation	Public functionary	352	12.1
	State-owned enterprise	873	30.1
	Private enterprise	260	9.0
	Government-sponsored institution	782	27.0
	Student	390	13.4
	Liberal professions	85	2.9
	Retirement	84	2.9
	Other	75	2.6

**Table 2:** The principal component analysis results for 17 items (*n* = 2901).

Perceived Constraints Factors	Sub-Constraints Factors	Factor Loadings	Eigen Value	Contribution Rate (%)	Cumulative (%)
Intrapersonal constraints	Physical problems	0.80	1.20	7.07	64.56
	Psychological problems	0.78			
	Negative attitudes toward fitness	0.76			
	Lack of interests	0.71			
Interpersonal constraints	Lack of partners	0.81	0.82	4.84	74.87
	Negative attitudes of family and friends	0.79			
Environmental condition constraints	Bad weather	0.73	0.93	5.48	70.04
	Fear of safety	0.75			
	Poor hygiene	0.71			
Leisure opportunity constraints	Lack of fitness fees	0.82	1.77	10.40	57.49
	Lack of time	0.80			
	Fitness activities are limited	0.77			
	Fitness fees are too high	0.73			
Facility-service management constraints	Lack of personalized services	0.81	8.01	47.09	47.09
	Lack of professional management	0.81			
	Inadequate programs and facilities	0.75			
	Lack of information	0.74			

**Table 3:** Results of the confirmatory factor analysis.

Variables (α)	M (SD)	Λ (Boot. SE)	z-Value	AVE	ρ
Intrapersonal constraints (α = 0.83)	4.02 (0.78)	0.53 (0.02)	23.30 ***	0.56	0.84
Physical problems	4.05 (0.94)	0.78 (0.01)	28.59 ***		
Psychological problems	3.83 (1.01)	0.79 (0.01)	27.64 ***		
Negative attitudes toward fitness	3.95 (3.95)	0.77 (0.01)	28.78 ***		
Lack of interests	4.23 (4.23)	0.66 (0.01)	33.33 ***		
Interpersonal constraints (α = 0.74)	3.75 (0.96)	0.68 (0.03)	19.82 ***	0.58	0.74
Lack of partners	3.82 (1.11)	0.75 (0.02)	23.04 ***		
Negative attitudes of family and friends	3.69 (1.06)	0.78 (0.02)	20.01 ***		
Environmental condition constraints (α = 0.79)	3.75 (0.92)	0.42 (0.03)	15.56 ***	0.61	0.82
Bad weather	3.49 (1.16)	0.56 (0.03)	35.87 ***		
Fear of safety	3.80 (1.09)	0.88 (0.01)	19.31 ***		
Poor hygiene	3.95 (1.04)	0.86 (0.01)	22.25 ***		
Leisure opportunity constraints (α = 0.90)	3.67 (0.93)	0.83(0.03)	26.55 ***	0.70	0.90
Lack of fitness fees	3.58 (1.10)	0.83 (0.01)	30.10 ***		
Lack of time	3.80 (1.07)	0.86 (0.01)	27.95 ***		
Fitness activities are limited	3.68 (1.04)	0.86 (0.01)	27.55 ***		
Fitness fees are too high	3.62 (1.03)	0.79 (0.01)	31.85 ***		
Facility-service management constraints (α = 0.91)	3.70 (0.88)	0.75 (0.03)	29.31 ***	0.72	0.91
Lack of personalized services	3.68 (0.99)	0.88 (0.01)	27.50 ***		
Lack of professional management	3.75 (0.98)	0.88 (0.01)	27.60 ***		
Inadequate programs and facilities	3.74 (1.01)	0.82 (0.01)	31.72 ***		
Lack of information	3.63 (1.01)	0.82 (0.01)	31.55 ***		
Active participation	3.88 (0.61)	—	—	—	—
Preference	4.19 (0.71)				
Frequency	3.57 (1.03)				

Note. C.R. = critical ratio; AVE = average variance explained; ρ = composite reliability; *** *p*-value < 0.001.

**Table 4:** Test for the convergent and discriminant validity of the latent constructs.

Construct	Correlation Coefficients				
Intrapersonal constraints	1				
Interpersonal constraints	0.67	1			
Environmental condition constraints	0.483	0.444	1		
Leisure opportunity constraints	0.569	0.573	0.627	1	
Facility-service management constraints	0.561	0.512	0.752	0.709	**1**

**Table 5:** Summary of the overall fit indices for the initial measurement model and final SEM model.

Overall	Absolute Fit Indices		Incremental Fit Indices		Parsimonious Fit Indices			
Fit Indices	GFI	RMSEA	AGFI	NFI	CFI	IFI	AIC	ECVI
Ideal indices	>0.9	<0.08	>0.9	>0.9	>0.9	>0.9	N/A	N/A
Initial measurement model	0.76	0.12	0.69	0.78	0.79	0.79	6683.48	0.35
Final structural model	0.96	0.05	0.95	0.97	0.97	0.97	1027.83	0.11

**Table 6:** Summary of the hypothesized SEM model (*n* = 2901).

The Hypothesized Paths	The Standardized Parameter Coefficients	Standard Errors	z-Value	Test Results
H1: Intrapersonal constraints→participation	−0.24	0.02	−4.40 ***	Acceptable
H2: Interpersonal constraints→participation	−0.22	0.02	−2.58 **	Acceptable
H3: Environmental condition constraints→participation	−0.004	0.02	−0.08	Not accepted
H4: Leisure opportunity constraints→participation	−0.28	0.01	−5.33 ***	Acceptable
H5: Facility-service management constraints→participation	−0.17	0.02	−3.31 ***	Acceptable

Note: *** *p*-value < 0.001, ** *p*-value < 0.01; For all analyses, *n* = 2901.

**Table 7:** Summary of the goodness-of-fit indices for the structural residuals model in multi-group comparisons of SEMs.

Overall Fit Indices	Ideal Indices	Inspection Results	Test Results
Absolute fit indices			
χ^2^	*p <* 0.05	5935.89 (*p* = 0.000 *<* 0.05)	Acceptable
RMSEA	<0.08	0.05	Acceptable
GFI	>0.90	0.90	Acceptable
AGFI	>0.90	0.90	Acceptable
Incremental fit indices			
NFI	>0.90	0.90	Acceptable
RFI	>0.90	0.90	Acceptable
IFI	>0.90	0.91	Acceptable
TLI(NNF1)	>0.90	0.90	Acceptable
CFI	>0.90	0.91	Acceptable
Parsimonious fit indices			
PGFI	>0.50	0.56	Acceptable
PNFI	>0.50	0.61	Acceptable
PCFI	>0.50	0.62	Acceptable
CN	>200	386	Acceptable
CMIN/DF	<2.00	17.06	Not accepted
AIC	The smaller the better	6379.89	Acceptable
ECVI	The smaller the better	1.10	Acceptable

## Data Availability

The data presented in this study are available on request from the corresponding author.
